# The Arabidopsis thylakoid chloride channel ClCe regulates ATP availability for light-harvesting complex II protein phosphorylation

**DOI:** 10.3389/fpls.2022.1050355

**Published:** 2022-11-22

**Authors:** Emilija Dukic, Peter J. Gollan, Steffen Grebe, Virpi Paakkarinen, Andrei Herdean, Eva-Mari Aro, Cornelia Spetea

**Affiliations:** ^1^ Department of Biological and Environmental Sciences, University of Gothenburg, Gothenburg, Sweden; ^2^ Molecular Plant Biology Unit, Department of Life Technologies, University of Turku, Turku, Finland; ^3^ Climate Change Cluster, University of Technology Sydney, Ultimo, NSW, Australia

**Keywords:** *Arabidopsis thaliana*, ATP synthase, chloride channel (ClC), light-harvesting complex II (LHCII), low light acclimation, photosystem II, protein phosphorylation, proton motive force (PMF)

## Abstract

Coping with changes in light intensity is challenging for plants, but well-designed mechanisms allow them to acclimate to most unpredicted situations. The thylakoid K^+^/H^+^ antiporter KEA3 and the voltage-dependent Cl^−^ channel VCCN1 play important roles in light acclimation by fine-tuning electron transport and photoprotection. Good evidence exists that the thylakoid Cl^−^ channel ClCe is involved in the regulation of photosynthesis and state transitions in conditions of low light. However, a detailed mechanistic understanding of this effect is lacking. Here we report that the ClCe loss-of-function in *Arabidopsis thaliana* results in lower levels of phosphorylated light-harvesting complex II (LHCII) proteins as well as lower levels of the photosystem I-LHCII complexes relative to wild type (WT) in low light conditions. The phosphorylation of the photosystem II core D1/D2 proteins was less affected either in low or high light conditions. In low light conditions, the steady-state levels of ATP synthase conductivity and of the total proton flux available for ATP synthesis were lower in ClCe loss-of-function mutants, but comparable to WT at standard and high light intensity. As a long-term acclimation strategy, expression of the *ClCe* gene was upregulated in WT plants grown in light-limiting conditions, but not in WT plants grown in standard light even when exposed for up to 8 h to low light. Taken together, these results suggest a role of ClCe in the regulation of the ATP synthase activity which under low light conditions impacts LHCII protein phosphorylation and state transitions.

## Introduction

Photosynthetic function is tightly coupled with growth and we need to understand how photosynthetic components are integrated to operate in a dynamic light environment. In low light, plants are limited in photosynthesis, and need mechanisms to maximize the light use efficiency. State transitions represent a reversible light acclimation mechanism for reconfiguring the photosynthetic light-harvesting apparatus to adjust excitation balance between photosystem II (PSII) and PSI and take place within a few minutes (Rantala et al., 2020). This is a way to maximize the efficiency of utilization of absorbed light energy under conditions when light is limiting for growth, by redistribution of a mobile pool of light-harvesting antenna (LHCII) trimers that can bind either to PSII (state 1) or to PSI (State 2). More specifically, in low light conditions favoring absorption by the PSII supercomplex, a part of LHCII (LHCB1 and LHCB2) are phosphorylated by the thylakoid bound STN7 kinase in the grana (Crepin and Caffarri, 2015). The phosphorylated LHCII (p-LHCII) dissociates from PSII, migrates to the grana margins and stroma lamellae where then associates with PSI, and functions as its light-harvesting antenna. Conversely, upon dephosphorylation, LHCII dissociates from PSI, migrates back to the grana and re-joins PSII. Noteworthy, while p-LHCB2 is essential for PSI-LHCI-LHCII complex formation (Crepin and Caffarri, 2015; 2018; Pan et al., 2018; Rantala et al., 2020), the p-LHCB1 is suggested to increase the mobility of PSII-LHCII supercomplexes and facilitate thylakoid remodelling (stacking, grana size) (Crepin and Caffarri, 2015). Importantly, a part of the mobile LHCII pool independently from phosphorylation is under most conditions associated with PSI (Croce, 2020). Grieco et al. (2015) brought evidence that, in addition to the phosphorylation-dependent movement of LHCII, entire complexes may migrate laterally to the grana margins in a LHCII antenna lake to allow for direct energy transfer from PSII to PSI under light that preferentially excites PSII. How LHCII and even large complexes like PSII and PSI move within the thylakoid membrane during state transitions is still unknown (Messant et al., 2021).

Three thylakoid-located ion channels/transporters, namely the thylakoid K^+^/H^+^ antiporter KEA3, the voltage-dependent Cl^−^ channel VCCN1 and the Cl^−^ channel ClCe have been unraveled in Arabidopsis to play important roles in the regulation of photosynthesis and light acclimation (Finazzi et al., 2015; Spetea et al., 2017; Szabo and Spetea, 2017). Deeper mechanistic understanding of how they work individually and in concert has evolved with the analyses of higher order loss-of-function mutants (Dukic et al., 2019; Hohner et al., 2019; Li et al., 2021). KEA3 and VCCN1 play a pivotal role in regulating the flow of K^+^, H^+^ and Cl^−^ ions across the thylakoid membrane, modulating the size and composition of the proton motive force (PMF), and in this way fine tuning photosynthetic electron transport and photoprotection, and indirectly many other chloroplast processes. The physiological role of ClCe is the least understood due to the weak photosynthetic phenotype of the Arabidopsis mutants (Dukic et al., 2019; Li et al., 2021). Nevertheless, ClCe loss-of-function in Arabidopsis altered the distribution of excitation energy between PSII and PSI during state transitions (Herdean et al., 2016a) and the electron transport in low light conditions (Dukic et al., 2019), but the mechanism remained unclear. Whether ClCe is a Cl^−^ channel or transporter is also not known due to unsuccessful heterologous expression. The activity as a Cl^−^/H^+^ exchanger for ClCf, which is the closest homologue of ClCe, has been demonstrated to be connected with the activity of the ATPase in Golgi membranes (Scholl et al., 2021). Here we aimed to decipher the mechanism behind altered state transitions in ClCe loss-of-function mutants by investigating if the LHCII phosphorylation and ATP synthase activity are affected. A compromised ATP synthesis is expected to perturb energy-dependent reactions such as LHCII protein phosphorylation with consequences for photosynthetic acclimation and plant growth. Based on analyses of *kea3, vccn1* and *clce* single, double, and triple mutants, we found lower levels of LHCII phosphorylation only in *clce* mutants and that this is caused by a reduced activity of the ATP synthase specifically under low light conditions. Moreover, the *clce* mutants displayed a lower photosynthetic performance and stronger shade avoidance traits relative to wild type when grown in light-limiting conditions.

## Materials and methods

### Plant material and growth conditions

*Arabidopsis thaliana* cv. Columbia-0 was used as wild type (WT). The mutant lines used in this study *clce-2 (c), kea3-1 (k), vccn1-1 (v), clce-2kea3-1 (ck), clce-2vccn1-1 (cv), kea3-1vccn1-1 (kv), and kea3-1vccn1-1clce-2* (*kvc*) were previously described (Dukic et al., 2019).

Plants used for phosphorylation experiments were grown in soil in growth chambers at 23°C and 60% relative humidity using 16 h light (100 µmol photons m^-2^ s^-1^)/8 h dark cycles (long-day) for 3 weeks or using 8 h light (120 µmol photons m^-2^ s^-1^)/16 h dark cycles (short-day) for 5 weeks. OSRAM PowerStar HQIT 400/D Metal Halide Lamps were used as light source. In some fluctuating light experiments, overnight dark-acclimated plants from short-day conditions were shifted to 15 µmol photons m^-2^ s^-1^ (LL1) conditions for 2 h, followed by 1000 µmol photons m^-2^ s^-1^ (HL) conditions for 2 h and back to 15 µmol photons m^-2^ s^-1^ (LL2) conditions for 2 h. Rosettes were harvested and frozen in liquid nitrogen after dark, 2 h in LL1, 2 h in HL, 15 min in LL2 and 2 h in LL2 conditions for later thylakoid isolations. Plants used for electrochromic shift (ECS) experiments were grown in soil for 6-8 weeks in a CLF PlantMaster chamber (Plant Climatics, Wertingen, Germany) using 8 h light (120 µmol photons m^−2^ s^−1^)/16 h dark cycles (short day) at 21°C/19°C, respectively, and 70% relative humidity. In some experiments, plants were grown using a short-day photoperiod in low light (15 µmol photons m^−2^ s^−1^) for up to 8 months.

### Thylakoid isolation and chlorophyll determination

Thylakoid isolations were performed with ice-cold reagents in cold room at 7°C. Fresh or frozen rosettes were ground in grinding buffer (50 mM HEPES–NaOH pH 7.5, 330 mM sorbitol, 5 mM MgCl_2_, 0.05% (w/v) BSA, 0.065% (w/v) Na-ascorbate and 10 mM NaF) and filtered through Miracloth. Chloroplasts were collected by centrifugation at 4000 *g* for 6 min at 4°C and ruptured osmotically in shock buffer (50 mM HEPES–NaOH pH 7.5, 5 mM MgCl_2_ and 10 mM NaF). Thylakoid membranes were collected by centrifugation at 4000 *g* for 6 min at 4°C and suspended in storage buffer (50 mM HEPES–NaOH pH 7.5, 100 mM sorbitol, 10 mM MgCl_2_, and 10 mM NaF). Chlorophyll (Chl) concentrations were determined in 80% (v/v) buffered acetone according to Porra et al. (1989).

### Gel electrophoresis and immunoblotting

For separation of native thylakoid protein complexes, large pore blue native polyacrylamide gel electrophoresis (lpBN-PAGE) was performed as described by Jarvi et al. (2011) with thylakoids solubilized in 1% (w/v) n-dodecyl-β-D-maltoside (DM) or 1% (w/v) digitonin (DIG). Additionally, thylakoid proteins were solubilized in Laemmli buffer (Laemmli, 1970) and separated by sodium dodecyl sulfate polyacrylamide gel electrophoresis (SDS-PAGE) in gels containing 15% (w/v) acrylamide and 6 M urea. For all gels, thylakoid samples were loaded on equal Chl basis.

For immunoblotting, proteins were transferred on PVDF membrane (Millipore) and recognized by specific antibodies for LHCB1 (1:5000, Agrisera), LHCB2 (1:5000, Agrisera), PsaB (1:3000, Agrisera), STN7 (1:1000, Agrisera), and CP47 (1:3000, gift from Prof. Roberto Barbato). Phosphorylated threonine residues were recognized with p-Thr antibody (1:3000, New England Biolabs) and phosphorylated LHCB1 (p-LHCB1, 1:10000, Agrisera) and LHCB2 (p-LHCB2, 1:10000, Agrisera). For detection, horseradish peroxidase-linked secondary antibody (Agrisera) and Amersham ECL Western blotting detection reagents (GE Healthcare) were used.

### RNA isolation and quantitative RT-PCR

Total RNA was isolated from plant tissues of 6-week-old plants with an E.Z.N.A. R6827-01 Plant RNA kit (Omega Bio-Tek, GA, USA) and residual DNA was removed with E1091 DNAse (Omega Bio-Tek). cDNA was synthesized using 500 ng of total RNA through iScript cDNA synthesis Kit (Bio-Rad, Hercules, CA, USA). Quantitative real-time PCR analyses were conducted with a SsoAdvanced Universal SYBR Green Supermix on a CFX96 Touch Thermal Cycler (Bio-Rad). 50 ng of cDNA was used as qPCR template in 10 µl reactions. Amplifications were done in two-step PCR with the following conditions: initial denaturation for 2 min at 95°C, followed by 40 cycles of denaturation for 5 s at 95°C, annealing for 30 s at 60° and extension for 10 s at 72°C. After amplification, melt-curve analyses were performed for all primers. Gene-specific primers used were ordered from Bio-Rad (**Supplementary Table 1**). ΔCq method (2^-ΔCq^) was used to calculate relative expression using *PEX4* and *ACTIN8* as reference genes.

### Kinetics of chlorophyll *a* fluorescence induction

*In vivo* Chl-*a* fluorescence in intact leaves of 30 min dark-acclimated plants was recorded using either a Handy-PEA, (Hansatech, UK) by applying saturating red actinic light of 3,500 µmol photons m^−2^ s^−1^ for 1 s or with a Dual-PAM 100 (Walz, Efeltrich, Germany) by applying a saturating pulse of 8,000 µmol photons m^-2^ s^-1^ for 700 ms and measuring light intensity<1.0 µmol photons m^-2^ s^-1^. Initial F_0_ and F_m_ fluorescence values were determined by the saturating pulse. The maximum quantum efficiency of PSII photochemistry (F_v_/F_m_) was calculated as ((F_m_-F_0_)/F_m_) according to Genty et al. (1989).

Slow kinetics of Chl fluorescence from the upper surface of leaves on intact plants were recorded using a closed FluorCam 800 MF (Photon System Instruments, Drasow, Czech Republic). Plants were acclimated in dark condition for 20 min before recording Chl fluorescence kinetics using the following settings: 6 min actinic white light at 15, 100 or 650 µmol photons m^−2^ s^−1^ followed by 2 min darkness, saturating flash of white light at 4,000 µmol photons m^−2^ s^−1^ for 800 ms applied after every min of light/dark, shutter speed 10 µs, and sensitivity 50%. In addition to the maximum quantum yield of PSII photochemistry in the dark-acclimated state (F_v_/F_m_), the PSII quantum yield (Y(II)) and the non-photochemical quenching (NPQ) at 6 min of illumination were calculated according to Genty et al. (1989). The photochemical quenching of PSII (fraction of open PSII reaction centers based on a lake model, qL), the quantum yield of regulated NPQ (Y(NPQ)), and the quantum yield of non-regulated energy dissipation (Y(NO)) were calculated as described (Kramer et al., 2004) using the minimum fluorescence in the light (F_0_′) estimated according to Oxborough and Baker (1997).

### Electrochromic band shift measurements

The proton motive force was estimated from ECS measured using the Dual-PAM 100 system equipped with a P515/535 module. Plants were first dark-acclimated for 30 min and then illuminated with actinic red light at 15, 100 or 650 µmol photons m^−2^ s^−1^ for 15 min. Before each measurement, three saturating single turnover 5-µs flashes at 200,000 µmol photons m^−2^ s^−1^ were applied to determine ECS_ST_, which was used to normalize ECS_t_. To determine the H^+^ conductivity of the ATP synthase (g_H_^+^), the light was switched off at specific time points to record the ECS signal decay during 620-ms dark intervals. The g_H_^+^ parameter was calculated as 1/τ (time constant for decay during the first 100 ms (Cruz et al., 2005). The total proton flux available for ATP synthesis was calculated as v_H_^+^ = PMF *×* g_H_^+^ (Cruz et al., 2001).

### CO_2_ fixation

Net rates of CO_2_ fixation were determined using a Li-COR Li-6400 (Lincoln, Nebraska, USA). Plants were first light acclimated in the growth chamber for at least 1 h and then exposed in the gas exchange chamber to broad spectrum light generated with red, green and blue LEDs of fixed intensity (30, 150 and 700 µmol photons m^−2^ s^−1^) in atmospheric CO_2_ (440 µmol mol^−1^) for approximately 5 min or until a steady state was reached. Since the leaf area was smaller than the chamber, data were normalized to leaf area determined using ImageJ.

### Leaf starch analysis

For visualization of starch accumulation and breakdown, leaves from plants grown in short day at 15 µmol photons m^−2^ s^−1^ were harvested at the end of the light cycle (end of the day) and end of the dark cycle (end of the night). All leaves were bleached in 80% (v/v) ethanol at 80°C for 10 min, and the starch content was visualized by Lugol’s staining for 10 min. After that, the leaves were rinsed and photographed on a light-table.

### Statistical analyses

Presented data are means ± S.E.M. for 3-6 plants. Statistical analyses to compare the means between two groups were performed using the Student’s *t*-test and among three or more groups using one-way ANOVA test. Statistically significant differences were considered at P< 0.05.

## Results

### LHCII and PSII core protein phosphorylation

**Figure 1:**
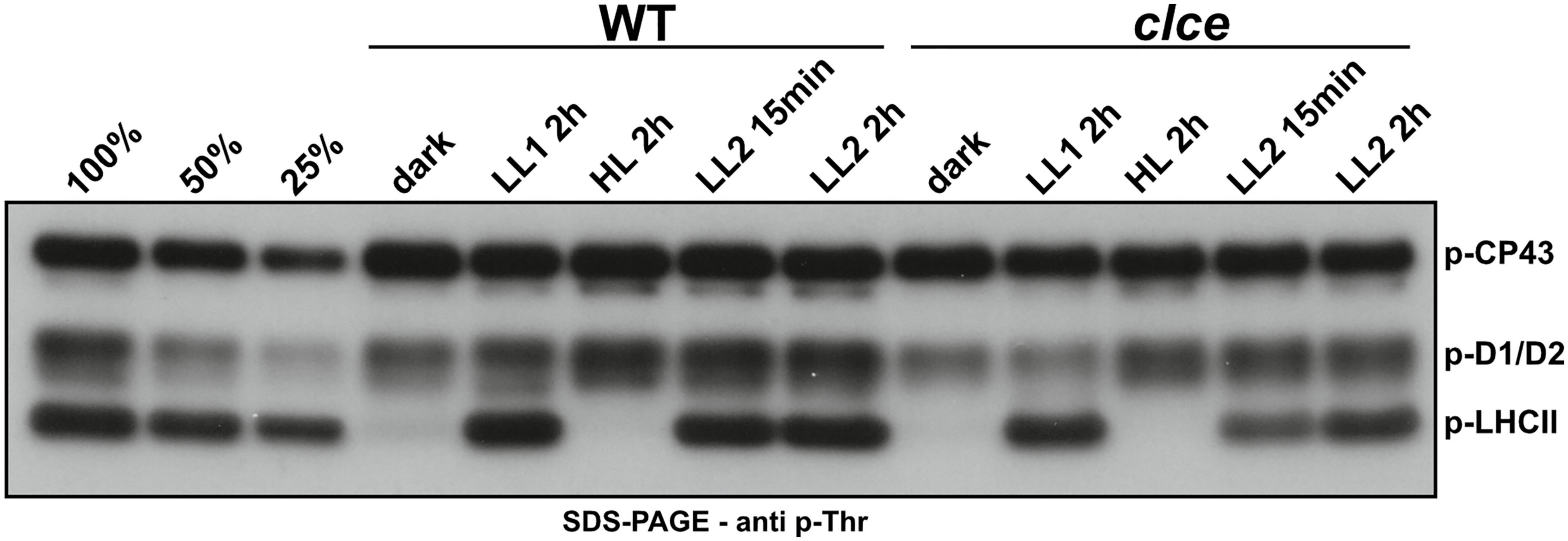
Phospho-Thr immunoblot of thylakoids from plants grown in short-day conditions. Wild-type plants (WT) and the *clce* mutant were grown using 8 h light (120 µmol photons m^−2^ s^−1^)/16 h dark cycles for 5 weeks. Overnight dark-acclimated plants were exposed to light at 15 µmol photons m^-2^ s^-1^ (LL1) for 2 h, followed by 1000 µmol photons m^-2^ s^-1^ (HL) for 2 h and back to 15 µmol photons m^-2^ s^-1^ (LL2) for 2 h. Thylakoids were isolated from leaves harvested at the given time points and light intensities. All thylakoids were loaded on equal chlorophyll basis of 0.5 µg. 100%, 50% and 25% WT sample after LL2 2h was used as loading control.

LHCII and PSII core proteins undergo reversible phosphorylation in higher plants in a light intensity-dependent manner. Shift to high-light intensity induces strong phosphorylation of the PSII core and decreases LHCII phosphorylation. Shift to lower-light intensity, in turn, decreases the phosphorylation of PSII core, but strongly induces the phosphorylation of LHCII (Rintamaki et al., 1997; 2000; Tikkanen and Aro, 2014). Therefore, we first investigated the phosphorylation pattern in WT and *clce* in different light conditions. Western blots of SDS-gels with a p-Thr antibody revealed lower levels of p-LHCII proteins in *clce* in low light conditions (as early as 15 min of illumination), but no difference from WT in high light conditions (**Figure 1**). The levels of p-D1/D2 proteins were only slightly lower as compared to WT in both low and high light.

**Figure 2:**
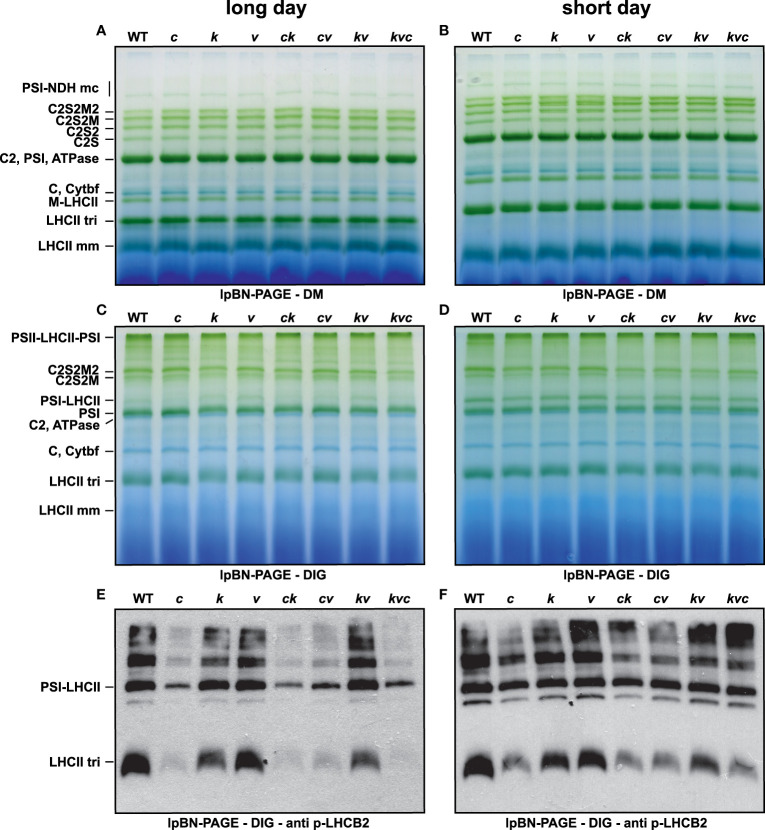
Levels of thylakoid protein complexes during long- and short-day conditions. Wild-type plants and mutants were grown using 16 h light (100 µmol photons m^−2^ s^−1^)/8 h dark cycles (long day) for 3 weeks **(A, C, E)** or 8 h light (120 µmol photons m^−2^ s^−1^)/16 h dark cycles (short day) for 5 weeks (**B, D, F**). **(A, B)** Large-pore blue-native gels (lpBN-PAGE) of thylakoids solubilized with 1% (w/v) n-dodecyl-β-D-maltoside (DM). **(C, D)** lpBN-PAGE of thylakoids solubilized with 1% (w/v) digitonin (DIG). **(E, F)** p-LHCB2 immunoblot from lpBN-PAGE of thylakoids solubilized with 1% (w/v) digitonin (DIG). Thylakoids were loaded on equal chlorophyll basis of 4 µg **(A–D)** and 1 µg **(E, F)**. WT – *Col-0*, *c* – *clce-2*, *k*– *kea3-1*, *v* – *vccn1-1*, *ck* – *clce-2kea3-1*, *cv* – *clce-2vccn1-1*, *kv* – *kea3-1vccn1-1*, and *kvc* – *kea3-1vccn1-1clce-2*.

Next, the WT plants and *clce* single, *clcevccn1, kea3clce, kea3vccn1* double, and *kea3vccn1clce* triple mutants were grown in either short- or long-day photoperiod. There was no visible difference in plant growth nor in the F_v_/F_m_ parameter among the genotypes (**Supplementary Figure 1**). From these plants, we analyzed the photosynthetic complexes from thylakoids solubilized with either DIG (preferentially solubilizes stroma thylakoids and preserves supramolecular interactions of the thylakoid protein complexes) or with DM (solubilizes protein complexes from the entire thylakoid membrane and breaks the most labile interactions). Lp-BN gels of DM-solubilized thylakoids revealed a minor increase in the levels of the various types of PSII complexes between WT and different mutants suggesting that the functional architecture of PSII remained largely unchanged (**Figures 2A–D**). Nevertheless, in thylakoids solubilized with DIG also the less abundant LHCII-PSI supercomplexes were detected in lp-BN gels and their abundance was reduced both by the ClCe mutation and by the length of the day used during plant growth. Western blots with an antibody against p-LHCB2 of such gels revealed overall lower amounts of p-LHCB2 isoform in native complexes only for the ClCe-lacking lines, especially in plants grown in long-day conditions (**Figures 2E, F**). These blots clearly revealed the impact of reduced LHCB2 phosphorylation on the abundance of the PSI-LHCII supercomplex, in line with previous reports (Crepin and Caffarri, 2015; Pan et al., 2018). Even though PSII-LHCII-PSI megacomplexes contained less p-LHCB2, their abundance was not affected, suggesting that the formation of PSII-LHCII-PSI megacomplexes is independent of phosphorylation (Yokono et al., 2015).

**Figure 3:**
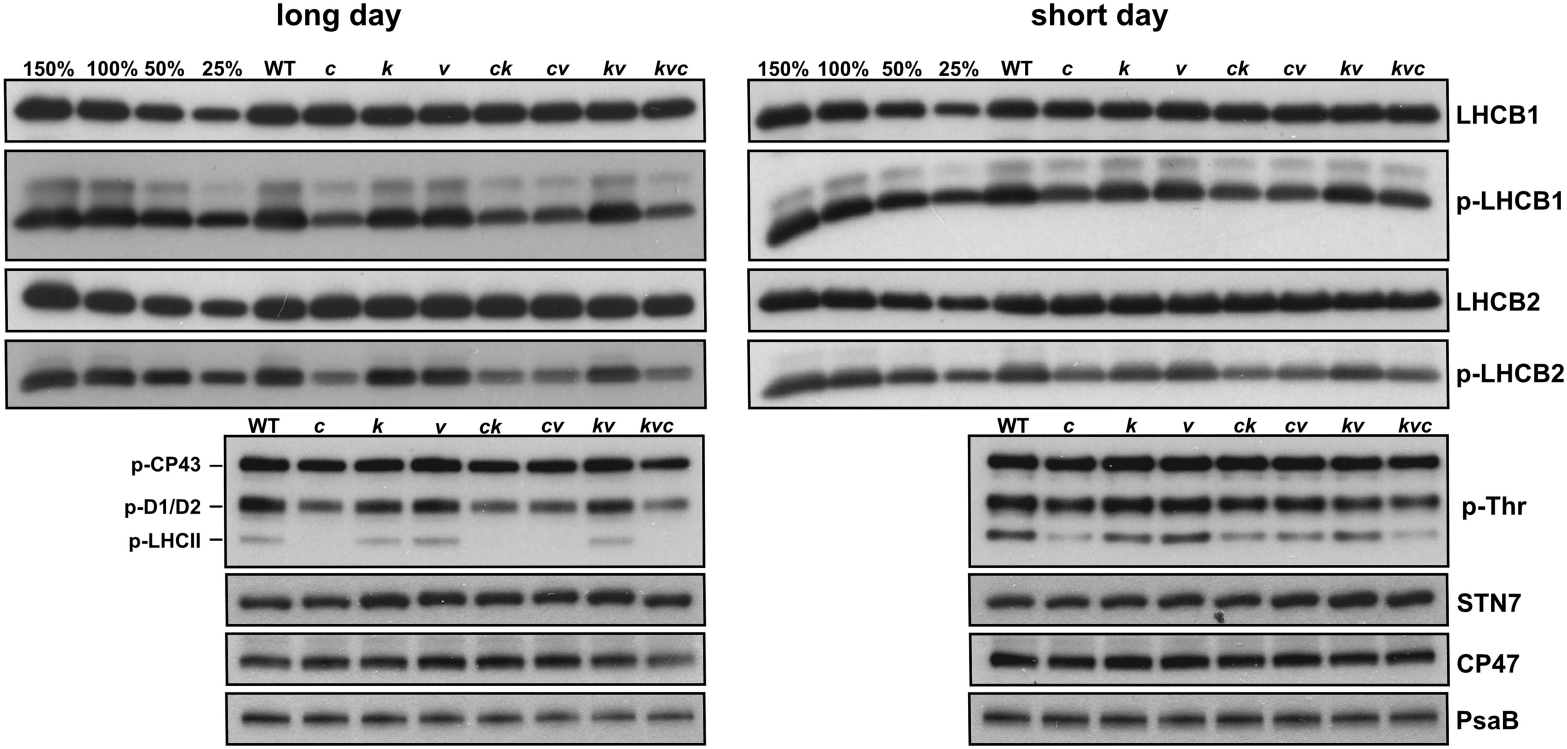
Thylakoid protein content and phosphorylation levels during long- and short-day conditions. Wild-type plants and mutants were grown using 16 h light (100 µmol photons m^−2^ s^−1^)/8 h dark cycles (long day) for 3 weeks or 8 h light (120 µmol photons m^−2^ s^−1^)/16 h dark cycles (short day) for 5 weeks. Immunoblots from thylakoids separated by SDS-PAGE and probed with LHCB1, p-LHCB1, LHCB2, p-LHCB2, p-Thr, STN7, CP47 (PSII) and PsaB (PSI) antibodies are shown. All thylakoids were loaded on equal chlorophyll basis of 0.5 µg, except 3 µg for STN7. 150%, 100%, 50% and 25% of WT samples were used as loading controls. WT – *Col-0*, *c* – *clce-2*, *k*– *kea3-1*, *v* – *vccn1-1*, *ck* – *clce-2kea3-1*, *cv* – *clce-2vccn1-1*, *kv* – *kea3-1vccn1-1*, and *kvc* – *kea3-1vccn1-1clce-2*.

Western blots presented in **Figure 3** show that the steady-state levels of p-LHCB1 and particularly those of p-LHCB2 were lower in the *clce* lines, and this was not due to altered levels of the corresponding proteins. Furthermore, the levels of the STN7 kinase, the PSII CP47 subunit as well as the PSI PsaB subunit showed similar abundance in WT and mutant plants grown in short-day conditions, but slight reductions in PSII and PSI in long-day photoperiod were observed in the *kea3clce* and *clcevccn1* double and the *kea3vccn1clce* triple mutants. Taken together, these data provide evidence that the ClCe loss-of-function results in decreased levels of LHCII protein phosphorylation relative to WT in low light conditions.

### Proton motive force, ATP synthase activity and CO_2_ fixation

**Figure 4:**
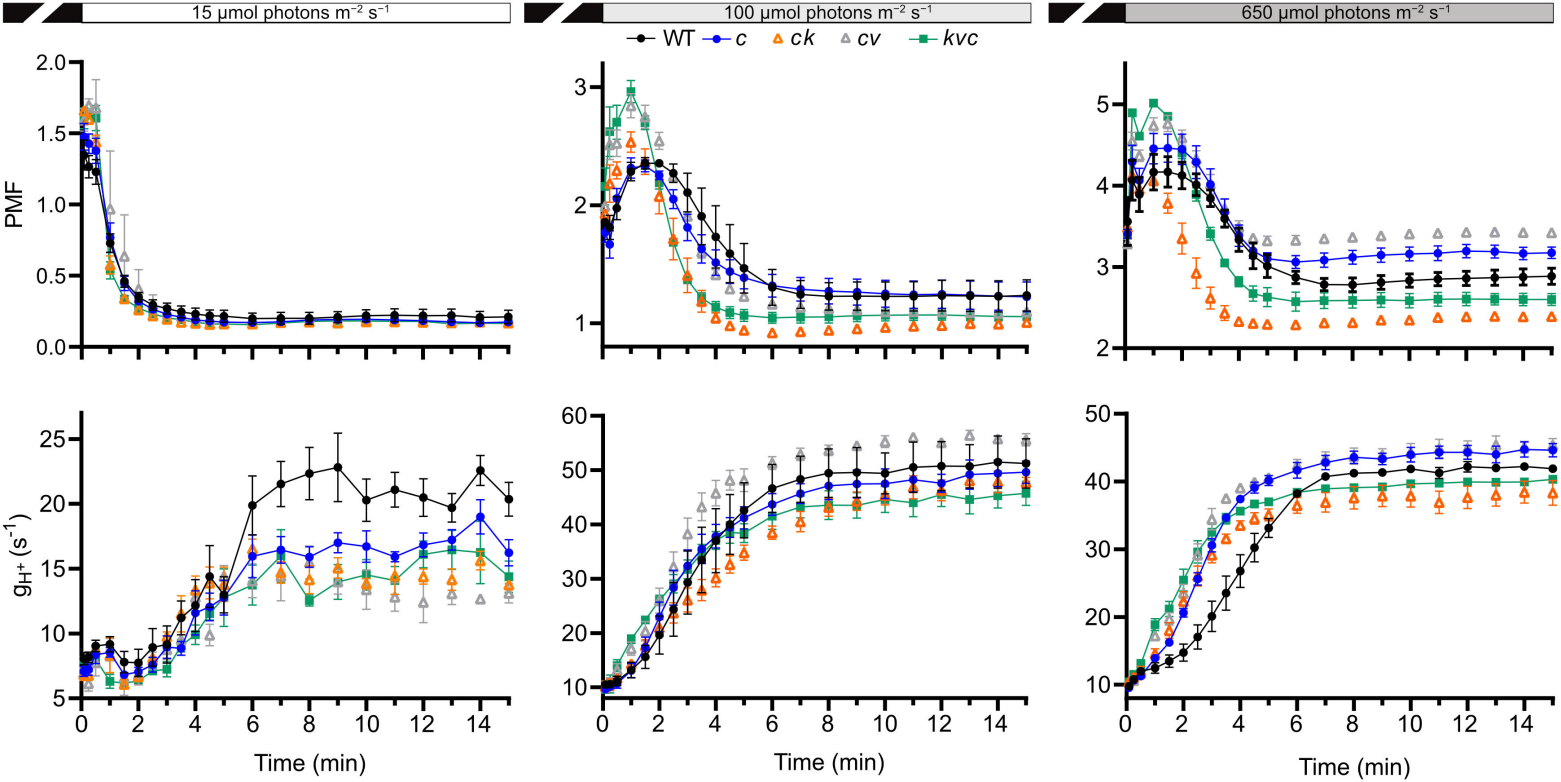
Kinetics of proton motive force and H^+^ conductivity through ATP synthase. Electrochromic shift measurements (ECS) were performed on 30 min dark-acclimated wild-type and mutant plants grown in short-day conditions (120 µmol photons m^−2^ s^−1^) and illuminated at the indicated intensities. Total proton motive force (PMF) and ATP synthase H^+^ conductivity (g_H_^+^) were calculated from ECS decay kinetics as described in Methods. The plotted data are means ± S.E.M. (*n* = 6 plants). WT – *Col-0*, *c* – *clce-2*, *k*– *kea3-1*, *v* – *vccn1-1*, and *kvc* – *kea3-1vccn1-1clce-2.* Induction kinetics for the first 210 illumination are presented in **Supplementary Figure 4**.

**Figure 5:**
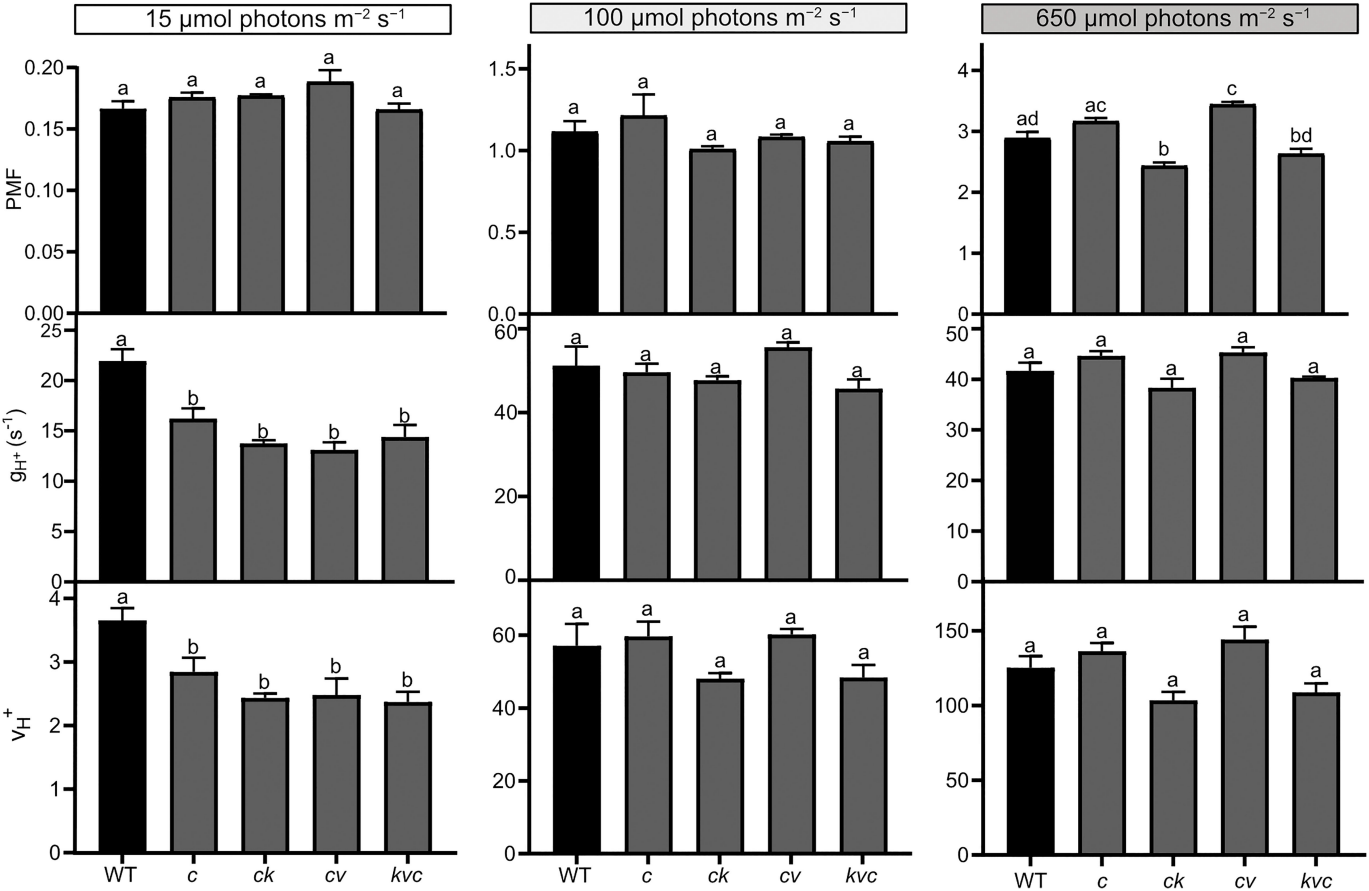
Steady-state proton motive force, H^+^ conductivity and H^+^ flux through the ATP synthase. Electrochromic shift measurements (ECS) were performed on 30 min dark-acclimated wild-type (WT) and mutant plants grown in short-day conditions (120 µmol photons m^−2^ s^−1^) and illuminated for 15 min at the indicated intensities. Total proton motive force (PMF), ATP synthase H^+^ conductivity (g_H_^+^) and H^+^ flux (v_H_^+^) were calculated from ECS decay kinetics as described in Methods. The plotted data were obtained from **Figure 4** and represent means ± S.E.M. (*n* = 6 plants). WT – *Col-0*, *c* – *clce-2*, *k*– *kea3-1*, *v* – *vccn1-1*, and *kvc* – *kea3-1vccn1-1clce-2.* Different letters denote statistically significant differences among genotypes according to ANOVA (*P*< 0.05).

LHCII protein phosphorylation requires energy from ATP produced by the ATP synthase on the thylakoid membrane. Since the abundances of the LHCII substrate and responsible STN7 protein kinase were not different among the genotypes, to explain the differences in LHCII phosphorylation observed in the *clce* mutants we explored if the activity of the ATP synthase was affected. To this aim, the plants were grown in short day at 120 µmol photons m^−2^ s^−1^ (**Supplementary Figure 2**) and subjected to ECS measurements during illumination for 15 min at 15, 100 and 650 µmol photons m^−2^ s^−1^, representing LL, GL, and HL conditions, respectively. From the ECS measurements, the thylakoid membrane total PMF size (estimated from ECSt), the conductivity to H^+^ of the ATP synthase (g_H_^+^) and the proton flux (v_H_^+^) were calculated. The g_H_^+^ parameter indicates the rate at which cations (mainly H^+^) move from the thylakoid lumen to the stroma mainly through the ATP synthase when briefly (100 ms) switching off the light. The ν_H_^+^ parameter takes into account both PMF and g_H_^+^ and thus provides the total flux of H^+^ available for ATP synthesis. Representative ECS recordings at two time points (210 s and 15 min) and three light intensities are shown for WT and *clce* in **Supplementary Figure 3**. Kinetics of PMF size and g_H_^+^ for the first 210 s are shown in **Supplementary Figure 4**. The complete kinetics are shown in **Figure 4**, and the steady-state values (at 15 min) in **Figure 5**.

LL was the condition where we observed a decreased level of LHCII phosphorylation in *clce* relative to WT (**Figure 1**). In LL we found a high total PMF at the onset of light up to 30 s in all genotypes (but slightly higher in the VCCN1-lacking mutants), then decreased and remained stable from 3 min until the end of illumination. There was no significant difference in PMF among the genotypes either at 210 s (**Supplementary Figure 4**, **Supplementary Table 2**) or 15 min (**Figure 4**). The g_H_^+^ parameter was low, stable, and similar among genotypes until 3 min (**Supplementary Figure 4**). The ATP synthase became gradually active up at 6 min and was fully functional until 15 min in WT (**Figure 4**). All ClCe-lacking mutants displayed a lower g_H_^+^ than WT during 6-15 min, suggesting a decreased ATP synthase activity. The plots in **Figure 5** more clearly show that while there was no significant difference in PMF size, the g_H_^+^ in *clce* was significantly lower as compared to WT (by 40%), and that additional mutations in KEA3 and VCCN1 did not interfere with these patterns. We have also plotted the v_H_^+^ at the last time point of illumination (15 min) showing significantly lower values in the *clce* mutants in LL conditions.

Lower levels of LHCII phosphorylation were also observed in *clce* mutants relative to WT in GL conditions (**Figure 3**). The PMF size in GL was high until 2-3 min (**Supplementary Figure 4**), declined until 6 min and remained stable until the end of illumination (**Figure 4**). PMF size did not differ between WT and the *clce* single line throughout the illumination. Nevertheless, PMF size was higher up to 2 min in VCCN1-lacking mutants (*clcevccn1* and *kea3 vccn1clce*), lower between 2-6 min in the lines lacking KEA3 (*kea3clce* and *kea3vccn1clce)* and reached WT levels in all genotypes by the end of illumination. The PMF size was significantly lower in KEA3-lacking lines at 210 s (**Supplementary Table 2**), and like WT at the end of illumination (**Figure 5**). By the time PMF size became low and stable (6 min), the g_H_^+^ raised and reached maximal steady state levels, which were not significantly different among the genotypes (**Figure 5**). Also, the steady state v_H_^+^ was like WT in all mutants in GL conditions (**Figure 5**).

Even though LHCII phosphorylation does not take place in HL, PSII core protein phosphorylation occurs under these conditions (**Figure 1**), prompting us to record ECS for comparison with LL and GL. All genotypes displayed a high PMF for 2-3 min (**Supplementary Figure 4**), declined until 6 min and remained stable until the end of illumination (**Figure 4**). However, the VCCN1-lacking lines (*clcevccn1* and *kea3 vccn1clce*) displayed higher PMF in the first min and the KEA3-lacking lines (*kea3clce* and *kea3 vccn1clce*) had lower PMF throughout the illumination. In fact, intermediate levels were observed in the triple mutant relative to *clcevccn1* and *clcekea3*. The g_H_^+^ was significantly higher in all *clce* mutants than in WT at the beginning of illumination (**Supplementary Figure 4**, **Supplementary Table 2**). The g_H_^+^ continued to be higher up to 6 min when it became stable and similar to WT (**Figure 4**). The observed discrepancies between differences in PMF and g_H_^+^ at HL indicate that additional mechanisms for H^+^ efflux across thylakoids may be functional in *clce* mutants under these light conditions before the ATP synthase becomes fully active. The lack of significant differences in g_H_^+^ and v_H_^+^ among the genotypes at 15 min (**Figure 5**) are in line with the similar PSII core phosphorylation levels observed as early as at 15 min of illumination (**Figure 1**). Taken together, our data suggest that specifically ClCe loss-of-function significantly slows down H^+^ efflux through the ATP synthase in the LL conditions, without impacting PMF size, and thus less ATP may become available for LHCII phosphorylation.

Inside the chloroplast, in addition to protein phosphorylation, ATP drives the highly energy-demanding CO_2_ fixation. To investigate if a deficit of ATP affected this process, plants grown at 120 µmol photons m^−2^ s^−1^ were illuminated at 30, 150 and 700 µmol photons m^−2^ s^−1^ for 5, 15, 30 min, 1 h, 2 h, 5 h and 8 h. We did not observe any differences in net photosynthesis (A_n_) among the genotypes at none of the tested light intensities and time points (**Supplementary Figure 5**), implying that the observed variation in H^+^ efflux through the ATP synthase in the *clce* lines (**Figure 5**) did not impact CO_2_ fixation. This result is in line with the WT-like growth of all mutants when grown in standard light (**Supplementary Figures 1**, **2**).

### Phenotypic analyses of plants grown in light-limiting conditions

Since the ATP synthase activity of *clce* was reduced in LL, the main emphasis was put on the growth and photosynthetic phenotype of plants grown in light-limiting conditions. The *clce* plants grown for 5-8 months at 15 µmol photons m^−2^ s^−1^ displayed smaller leaves with elongated petioles and significantly longer stem as compared to WT (**Supplementary Figures 6A–E**). The elongated stem likely represents a shade avoidance trait that plants normally develop when growing in limiting light (Franklin, 2008). Even though also observed in WT, this growth phenotype was more pronounced in the *clce* mutant, likely as a trade-off to get closer to the light source and absorb more light. The maximum PSII efficiency in the dark-acclimated state measured with Handy-PEA was slightly but significantly lower in *clce* than in WT (**Supplementary Figure 6F**).

Due to the small leaf size of plants grown at 15 µmol photons m^−2^ s^−1^, we could not perform Chl fluorescence induction nor ECS kinetics in the light-acclimated state with the Dual-PAM 100. Instead, we used an Imaging FluorCam and further assessed the photosynthetic performance of *clce* plants relative to WT at 6 min of illumination at 15, 100 and 650 µmol photons m^−2^ s^−1^. The maximum PSII efficiency in the dark state (F_v_/F_m_) was significantly lower in *clce* as compared to WT (**Table 1**) in line with the data obtained with Handy-PEA (**Supplementary Figure 6F**). During illumination, PSII efficiency (Y(II)) decreased with increasing light intensity and was significantly lower in *clce* than in WT at 15 µmol photons m^−2^ s^−1^. The yield of non-regulated energy dissipation (Y(NO)) increased with increase in light intensity and was significantly higher in *clce* than in WT at 15 µmol photons m^−2^ s^−1^. The yield of regulated NPQ (Y(NPQ)) increased with increasing light intensity and was not significantly different between WT and *clce*. Similar results were obtained for the NPQ parameter. Estimation of the fraction of open PSII centers, qL (Kramer et al. (2004), showed a clear decline with increase in light intensity and was significantly lower in *clce* compared to WT at 15 µmol photons m^−2^ s^−1^.

**Table 1:** Chlorophyll fluorescence parameters of plants grown in light-limiting conditions.

Parameter	15 µmol photons m^-2^ s^-1^		100 µmol photons m^-2^ s^-1^		650 µmol photons m^-2^ s^-1^	
	WT	*clce*	WT	*clce*	WT	*clce*
F_v_/F_m_	0.823 ± 0.003	0.802 ± 0.005*				
Y(II)	0.741 ± 0.017	0.712 ± 0.014*	0.282 ± 0.035	0.312 ± 0.077	0.158 ± 0.044	0.137 ± 0.019
Y(NO)	0.216 ± 0.018	0.249 ± 0.024*	0.364 ± 0.019	0.353 ± 0.019	0.410 ± 0.019	0.408 ± 0.027
Y(NPQ)	0.043 ± 0.008	0.039 ± 0.023	0.354 ± 0.029	0.335 ± 0.061	0.432 ± 0.028	0.456 ± 0.019
NPQ	0.199 ± 0.043	0.165 ± 0.097	0.977 ± 0.096	0.944 ± 0.143	1.055 ± 0.073	1.124 ± 0.144
qL	0.693 ± 0.048	0.607 ± 0.035*	0.161 ± 0.025	0.210 ± 0.062	0.099 ± 0.028	0.091 ± 0.012

Wild type (WT) plants and the *clce* mutant were grown in short-day conditions at 15 µmol photons m^-2^ s^-1^ for 8 months. Plants were acclimated for 20 min in darkness and then illuminated at the given intensities for 6 min in a closed FluorCam. Chlorophyll fluorescence was recorded during illumination and the following parameters were calculated as described in Methods: F_v_/F_m_ – the maximum PSII quantum yield, Y(II) – the PSII quantum yield in the light, Y(NO) – the quantum yield of non-regulated energy dissipation, Y(NPQ) – the quantum yield of regulated non-photochemical quenching, NPQ – non-photochemical quenching, and qL – the fraction of open PSII centers based on a lake model. Data are the means ± S.E.M. (n ≥ 4 leaves). Asterisks denote statistically significant differences between WT and *clce* according to Student’s t-test (P< 0.05).

Since assimilated CO_2_ is transiently stored as starch in leaf chloroplasts during the day and is broken down during the night to supply energy for metabolism and growth, we next investigated the starch accumulation at the end of the day and night in WT plants and the *clce* mutant grown at 15 µmol photons m^−2^ s^−1^. As shown in **Supplementary Figure 7A**, higher levels of starch (darker coloration) accumulated in the *clce* at the end of the light cycle. Moreover, *clce* also showed less depletion of starch at the end of the dark period relative WT (**Supplementary Figure 7B**), indicating decreased rates of breakdown. Comparing the dark and light periods, starch was not at all degraded during the night in *clce*, whereas there was clearly more starch in light than in dark in the WT. Taken together, these data provide evidence that the ClCe loss-of-function in plants grown in light limiting conditions results in decreased photosynthetic performance, altered starch metabolism and growth.

### ClCe expression levels and upregulation of other genes

**Figure 6:**
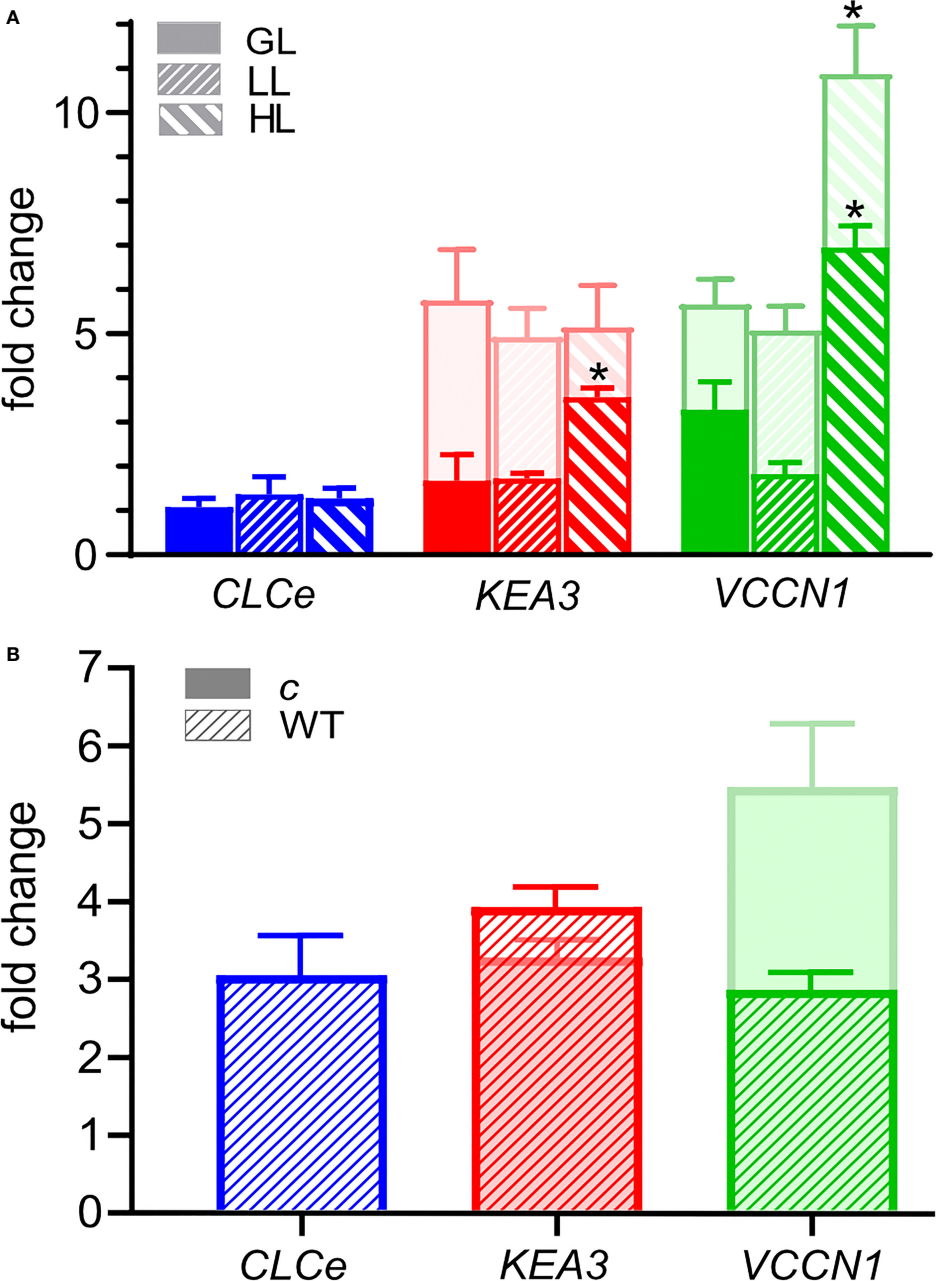
Fold change in the expression of *CLCe*, *KEA3* and *VCCN1* genes. **(A)** Wild type plants (WT, front bars) and *clce* mutants *(*bars behind, lighter color) were grown in short-day conditions with 8 h light (120 µmol photons m^−2^ s^−1^)/16 h dark for 6 weeks. Total RNA was isolated after 16-h dark and 3 h exposure to low light (LL, 15 µmol photons m^−2^ s ^−1^), growth light (GL, 120 µmol photons m^−2^ s ^−1^) or high light (HL, 650 µmol photons m^−2^ s^−1^), and changes in transcript abundance were determined by quantitative RT-PCR analysis. The expression of *CLCe, KEA3* and *VCCN1* genes was calculated relative to two reference genes and normalized to expression in samples collected after the 16-h of dark period. Data are the means ± S.E.M. (*n* = 4). Asterisks denote a statistically significant difference between each of the light treatments and the dark control according to Student’s *t*-test (*P*< 0.05). **(B)** WT and *clce* plants were grown in short-day LL condition (15 µmol photons m^−2^ s^−1^) for 8 months. Total RNA was isolated after 16-h dark and 3 h exposure to LL and changes in transcript abundance were determined by quantitative RT-PCR analysis. The relative expression of *CLCe, KEA3* and *VCCN1* genes was calculated as in **(A)**. Data are the means ± S.E.M. (*n* = 4).

We also investigated if *ClCe, KEA3* and *VCCN1* gene expression changes in WT versus *clce, kea3* and *vccn1* single mutants grown in standard GL light and exposed at three intensities (GL, LL and HL) over a time course of 3 and 8 h. The relative expression of *CLCe* did not change in WT over time nor in different light conditions versus darkness (**Figure 6A** and **Supplementary Figure 8A**). *KEA3* expression in WT did not differ between LL and GL relative to darkness, but it was significantly higher at 3 h in HL (3-fold), while *KEA3* expression in *clce* was upregulated to similar levels in comparison to darkness (5-fold) at all three light intensities (**Figure 6A** and **Supplementary Figures 8A, B**). *VCCN1* expression in WT did not significantly differ between LL and GL relative to darkness, however HL significantly upregulated it (6-fold, **Figure 6A**). In *clce*, the expression of *VCCN1* was upregulated 5-fold in LL and GL relative to darkness and 10-fold in HL (**Figure 6A**). The expression of *ClCe* was not altered relative to darkness in *kea3* and *vccn1* mutants at any of the three studied light intensities (**Supplementary Figures 8C, D**). The expression of *VCCN1* gene was upregulated after 8 h in HL in the *kea3* mutant, and of *KEA3* expression in the *vccn1* mutant.

In plants grown at 15 µmol photons m^−2^ s^−1^, the expression of *ClCe* in WT leaves was upregulated 3-fold relative to darkness (**Figure 6B**), supporting the importance of ClCe in this light condition. The upregulation of *CLCe* expression may be a long-term acclimation strategy during growth in light-limiting conditions since no such effect was observed in WT leaves after up to 8 h exposure to LL (**Figure 6A**). The expression of *KEA3* gene was similar in WT and *clce* (3-4-fold upregulation versus darkness), whereas *VCCN1* expression was higher in *clce* (5-fold) versus WT (3-fold) (**Figure 6B**). Notably, when comparing the fold changes in **Figures 6A, B**, the upregulation of *KEA3* and *VCCN1* in *clce* was maximal in LL already at 3 h, since no large changes happened during growth in such light-limiting conditions.

## Discussion

ClCe functions in the thylakoid membrane where also VCCN1 and KEA3 are active, although in different light conditions and with different kinetics. KEA3 and VCCN1 are particularly important in photosynthetic acclimation in the first minutes of constant illumination and under fluctuating light conditions (Dukic et al., 2019; Li et al., 2021). Good evidence exists that ClCe regulates electron transport through photosystems during illumination in constant light later in time than VCCN1 and KEA3 (Dukic et al., 2019) and during state transitions (Herdean et al., 2016a), but the mechanism has remained unclear. Previous studies investigating KEA3 (Correa Galvis et al., 2020), VCCN1 (Herdean et al., 2016b) and ClCe (Herdean et al., 2016a) found that VCCN1 and ClCe affected the PMF and the ATP synthase activity in high light conditions. Here we show that ClCe regulates ATP availability for LHCII phosphorylation and in this way maximizes photosynthetic performance particularly under light-limiting conditions.

### Role of ClCe in the regulation of electron transport and ATP production

The growth and F_v_/F_m_ of *clce* in standard light (100-120 µmol photons m^−2^ s^−1^) were like WT (**Supplementary Figures 1**, **2**). When grown in limiting light (15 µmol photons m^−2^ s^−1^), there were differences in growth (**Supplementary Figure 6**) and slightly, but significantly lower F_v_/F_m_, lower Y(II) and higher Y(NO) for *clce* plants (**Table 1**), suggesting that ClCe plays a role in regulation of electron transport in low light. We could not perform ECS measurements due to the small leaf size of these plants. Nevertheless, when plants grown in standard GL light were exposed for 10 min to LL, the g_H_^+^ as well as v_H_^+^ of *clce* significantly decreased (by 40%) relative to WT (**Figure 5**). The total PMF which drives ATP synthesis did not differ between WT and mutants, excluding the PMF size as a cause for the observed reduction in g_H_^+^. We do not know at present how the overall higher relative expression of *VCCN1* and *KEA3* in the *clce* mutant than in WT (**Figure 6**) could account for the observed differences in PMF and g_H_^+^ (**Figure 5**). Moreover, additional mutations in KEA3 and VCCN1 did neither enhance or reduce the effect on g_H_^+^ and v_H_^+^ in *clce*, suggesting that ClCe alone is a regulator of ATP synthase activity in low light conditions.

To dissect the mechanism behind the effect on ATP synthase activity, it is necessary to consider its possible regulators in low light. Kohzuma et al. (2017) proposed that the PMF generated already at very low light intensity (10 µmol photons m^-2^ s^−1^) is far above to what is needed to activate the ATP synthase, suggesting that PMF acts more as a dark-light switch rather than a finetuning mechanism. Another important modulator is the NADPH-dependent thioredoxin reductase C (NTRC) affecting the redox state of the ATP synthase γ-subunit thiols (Nikkanen et al., 2016). Like *clce*, the *ntrc* mutants had a lower g_H_^+^ than WT in low light, but in contrast to *clce*, *ntrc* displayed a high PMF, higher NPQ and lower electron transport rate than WT (Carrillo et al., 2016), excluding that the NTRC activity was affected in our mutants. Kanazawa and Kramer (2002) postulated that the g_H_^+^ is modulated by stromal metabolite levels, possibly by inorganic phosphate (Pi). Indeed, decreases in g_H_^+^ were reported in Pi deprivation and thylakoid Pi transporter PHT4;1 loss-of-function and were associated with high NPQ and low electron transport rates (Karlsson et al., 2015; Carstensen et al., 2018). However, we did not see any evidence of Pi limitation in our growth conditions. We postulate that H^+^ are supplied to the ATP synthase by a H^+^/Cl^−^ exchange activity of ClCe to explain its role in g_H_^+^ regulation.

Phylogenetic analysis of the ClC family indicated that the ClCe sequence is highly similar to bacterial ClCs, shown to function as H^+^/Cl^−^ antiporters, and that its closest homologue is ClCf (Pfeil et al., 2014). Also, animal ClCs function as H^+^/Cl^−^ exchangers in organellar membranes while they work as Cl^–^ channels at the plasma membrane (Jentsch, 2015). Since three selectivity filter residues important for H^+^ (gating glutamate, proton glutamate) and Cl^–^ binding (Schrecker et al., 2020) are fully conserved in ClCe and ClCf, they are likely also working as exchangers (Scholl et al., 2021). ClCf is located on trans Golgi network, a compartment involved in protein transport to their destination. Here ClCf works together with the V-ATPase to maintain pH and ion homeostasis. By analogy, in chloroplasts a concerted action of ClCe and the ATP synthase may be required to maintain the optimal pH in low light. Like other ClCs, the amino acid sequence of ClCe consists of a transmembrane component for ion transport and a regulatory cystathionine beta-synthase (CBS)-pair domain which binds nucleotides (Dutzler et al., 2002). The ATP pool may vary according to the photosynthetic status of the chloroplast in different light conditions. We propose that the CBS domain is sensitive to a low ATP concentration in low light and activates ClCe to export Cl^–^ to stroma and import H^+^ into the thylakoid lumen which in turn stimulates ATP synthesis.

A reduced ATP synthase activity in low light should impact various energy-dependent processes in the chloroplast. Indeed, *clce* displayed reduced starch degradation (**Supplementary Figure 7**), a process occurring in darkness and with high demand of ATP for the first phase taking place inside the chloroplast (Stitt and Zeeman, 2012). Since we observed no effect on carbon fixation (**Supplementary Figure 5**), we focus below our discussion on the LHCII phosphorylation that was most affected, and on its consequence for state transitions in low light conditions.

### Role of ClCe in LHCII protein phosphorylation and state transitions

Among the studied thylakoid ion channels/transporters, a role in state transitions was proposed for ClCe (Herdean et al., 2016a). The envelope located KEA1 and KEA2 have been also studied, but no differences in either LHCII phosphorylation or state transitions could be found in the corresponding mutants (Koskela et al., 2018). Here we show a decreased level of LHCII phosphorylation in *clce* in low light (**Figure 1**). Among the factors that could be responsible for this effect, we exclude changes of the levels of protein substrates (LHCB1 and LHCB2) and of the involved STN7 kinase as they were unaltered in *clce* (**Figure 3**). The redox state of the PSII acceptor side, based on the qL parameter, was significantly more reduced in *clce* than in WT in low light, likely leading to an increase in non-regulated energy dissipation, Y(NO) (**Table 1**). This fluorescence phenotype resembles the reported increase in steady-state fluorescence levels (F/F_m_) in mutants lacking STN7 (Grieco et al., 2012; Tikkanen et al., 2017), thus supporting the notion of affected state-transitions. The only conceivable factor for the scarcity of LHCII phosphorylation is the ATP availability which was lower in *clce* as based on the 40% reduced g_H_^+^ and v_H_^+^ (**Figure 5**). Availability of ATP as a driving force of state transitions in low light has not been emphasized previously, but in this study, we bring strong evidence for this type of regulation mediated by ClCe. A lower level of LHCII phosphorylation in *clce* was also observed in GL conditions (**Figure 3**). Since neither ATP synthase activity nor Y(NO) were significantly different, other factors could have prevented accumulation of phosphorylated LHCII in GL conditions.

For a long time, phosphorylation of LHCII was the main factor required for migration of LHCII during state transitions. Based on the decreased levels of LHCII phosphorylation, a slower migration would be expected in *clce*. However, Herdean et al. (2016a) reported a faster transition from state 1 to state 2 in this mutant as compared to WT, meaning a faster migration of LHCII from PSII to PSI. It should be noted that the rate of state transitions is also affected by the antenna composition of PSII (Kovacs et al., 2006) and PSI (Damkjaer et al., 2009). However, since KCl-pretreatment partially alleviated the faster state transition in *clce*, other factors like negative charges, could also influence the migration. Lack of the NUCLEAR SHUTTLE INTERACTING (NSI), enzyme for Lys acetylation of specific PSI, PSII, and LHCII subunits, slowed down state transitions despite similar levels of LHCII phosphorylation (Koskela et al., 2018). The authors proposed that a deficit of negative charges on the stromal side inhibited LHCII migration from PSII to PSI. Faster state transitions were reported in loss of function of the PsbW protein, known to harbor a stromal-exposed C-terminus rich in negative charges (Garcia-Cerdan et al., 2011). It can be reasoned that *clce* must have an excess of negative charges inside the lumen, leading to enhanced mobility and migration of LHCII in the membrane despite the decreased phosphorylation levels. Since KCl-pretreatment of leaves slowed down state transitions (even in WT) (Herdean et al., 2016a), this treatment likely balanced the distribution of negative charges on the two sides of the thylakoid membrane. We can conclude that while phosphorylation facilitates a more controlled LHCII migration, other factors increasing the negative charges of the LHC proteins also regulate state transitions.

Mutations in genes encoding ClCs lead to physiological disorders, including severe diseases in humans. The results of our phenotypic analyses reveal that ClCe regulates ATP availability for LHCII protein phosphorylation in state transitions. Since ClCe function is important for plant acclimation to low light, more in-depth studies are required for future applications towards crop improvement.

## Data availability statement

The original contributions presented in the study are included in the article/**Supplementary Material**. Further inquiries can be directed to the corresponding author.

## Author contributions

ED, PJG, AH, E-MA, and CS conceived the study and designed the experiments. ED carried out the ECS, Chl fluorescence and CO_2_ fixation measurements, starch staining and qRT-PCR. SG contributed to the analysis and interpretation of the Chl fluorescence data. VP and PJG carried out the phosphorylation experiments and immunoblotting. ED, SG, E-MA, and CS wrote the manuscript. All authors contributed to the article and approved the submitted version.

## Funding

This work was supported by the Swedish Research Council VR 2016-03836 and 2021-03790 (CS), and Jane and Aatos Erkko Foundation (E-MA).

## Conflict of interest

The authors declare that the research was conducted in the absence of any commercial or financial relationships that could be construed as a potential conflict of interest.

## Publisher’s note

All claims expressed in this article are solely those of the authors and do not necessarily represent those of their affiliated organizations, or those of the publisher, the editors and the reviewers. Any product that may be evaluated in this article, or claim that may be made by its manufacturer, is not guaranteed or endorsed by the publisher.

## Abbreviations

Chl, chlorophyll; ClC, chloride channel; DIG, digitonin; DM, n-dodecyl-β-D-maltoside; F_v_/F_m_, maximum quantum yield of photosystem II photochemistry; GL, growth light; HL, high light; LEF, linear electron transport; LHC, light harvesting complex; LL, low light; NPQ, non-photochemical quenching; g_H_^+^, proton conductivity through ATP synthase; v_H_^+^, proton flux; PMF, proton motive force; PS, photosystem; qL, fraction of open PSII centers based on a lake model; Y(II), quantum yield of PSII photochemistry; Y(NO), quantum yield of non-regulated energy dissipation; Y(NPQ), quantum yield of regulated non-photochemical quenching.
