# Supplementary material for: The Arabidopsis thylakoid chloride channel ClCe regulates ATP availability for light-harvesting complex II protein phosphorylation
Source: Front Plant Sci. 2022 Nov 22;13:1050355. doi: 10.3389/fpls.2022.1050355 (PMC9722747; doi:10.3389/fpls.2022.1050355)
Supplement: Supplementary file 1 [file DataSheet_1.pdf]

Supplementary Material

The Arabidopsis thylakoid chloride channel ClCe regulates ATP availability for light-harvesting complex II protein phosphorylation

Emilija Dukic[1], Peter J. Gollan[2], Steffen Grebe[2], Virpi Paakkarinen[2], Andrei Herdean[3], Eva-Mari Aro[2] and Cornelia Spetea[1*]

[1]Department of Biological and Environmental Sciences, University of Gothenburg, Gothenburg, Sweden

[2]Molecular Plant Biology Unit, Department of Life Technologies, University of Turku, Turku, Finland

[3]Climate Change Cluster, University of Technology Sydney, Ultimo, Australia.

*** Correspondence:**
Corresponding Author
cornelia.spetea.wiklund@bioenv.gu.se

Supplementary Figures

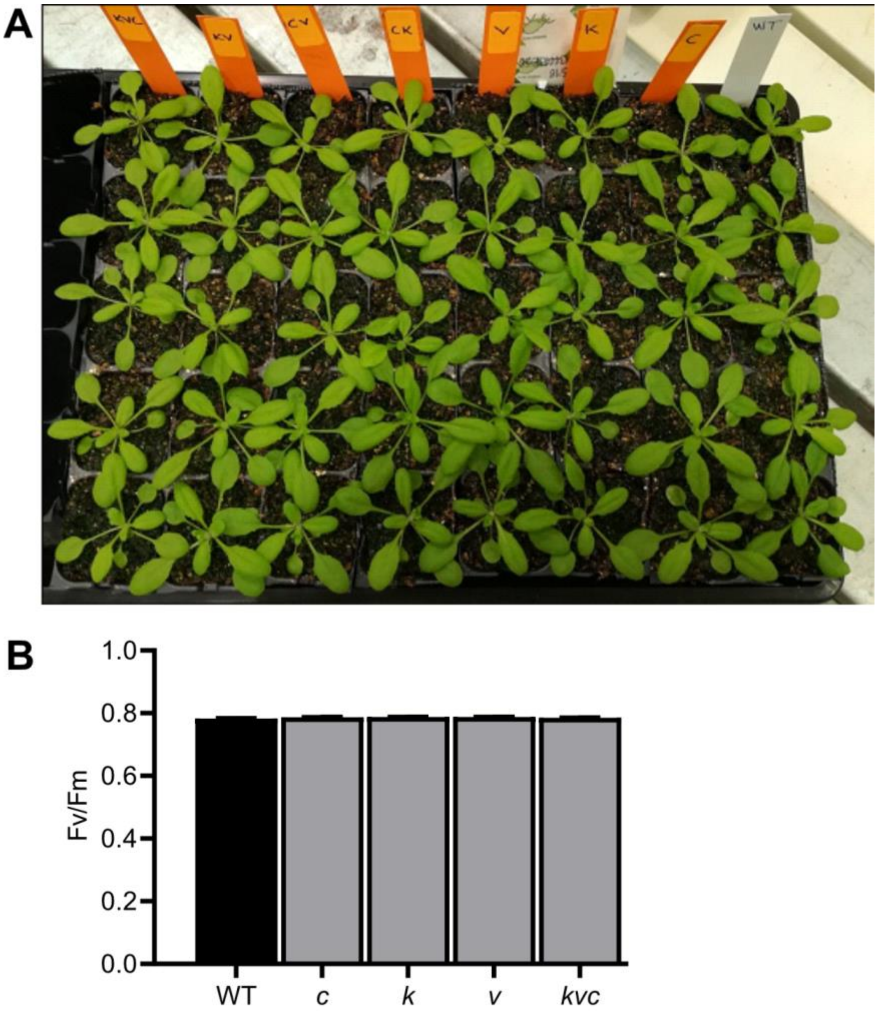

Supplementary Figure 1. Growth phenotype and F_v/F_m of long-day grown plants in the Turku laboratory. **(A)** Representative photos of wild-type plants and mutants grown using 16-h light (100 µmol photons m^{-2} s^{-1})/8 h dark cycles for 3 weeks. (**B**) F_v/F_m was determined on leaves from 30 min dark-acclimated plants. Data are the means ± S.E.M. (*n* = 6-10 plants). There were no statistically significant differences among genotypes at any of the tested conditions according to ANOVA ($P > 0.05$). WT – *Col-0*, *c* – *clce-2*, *k*– *kea3-1*, *v* – *vccn1-1*, and *kvc* – *kea3-1vccn1-1clce-2*.

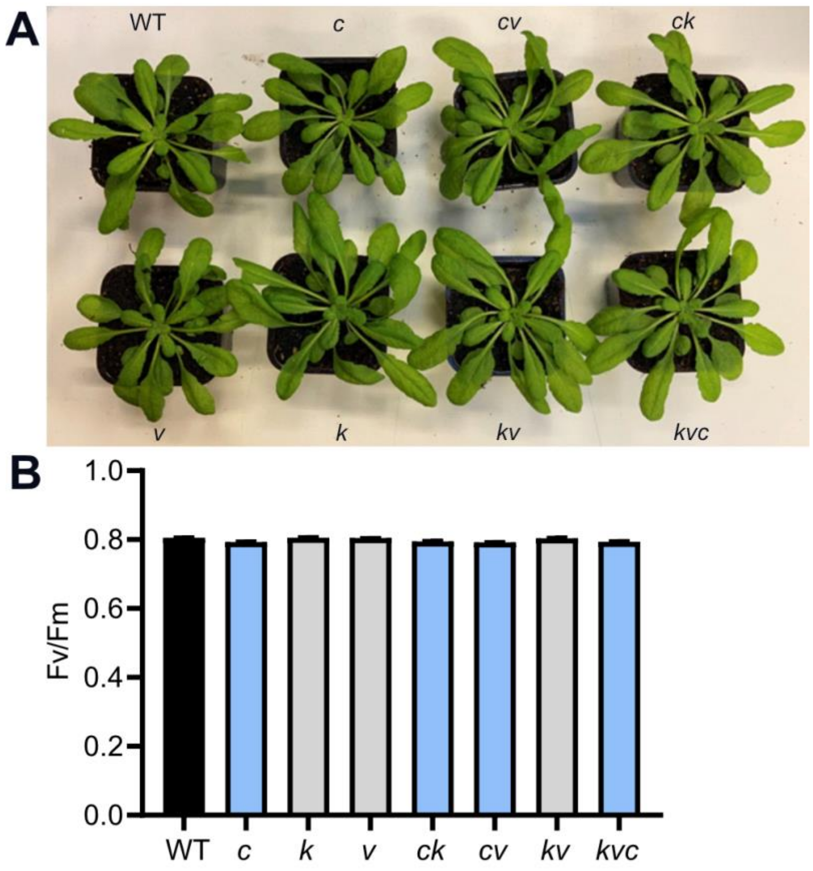

Supplementary Figure 2. Growth phenotype and F_v/F_m of short-day grown plants in the Gothenburg laboratory. Wild-type plants and mutants were grown using 8 h light (120 µmol photons m^{-2} s^{-1})/16 h dark cycles for 6-8 weeks. (**A**) Representative photos of plants are shown. (**B**) F_v/F_m was determined on 30 min dark-acclimated plants. Data are the means ± S.E.M. (n = 7-10 plants). There were no statistically significant differences among genotypes at any of the tested conditions according to ANOVA ($P > 0.05$). WT – *Col-0, c – clce-2, k– kea3-1, v – vccn1-1, ck – clce-2kea3-1, cv – clce-2vccn1-1, kv – kea3-1vccn1-1*, and *kvc – kea3-1vccn1-1clce-2*.

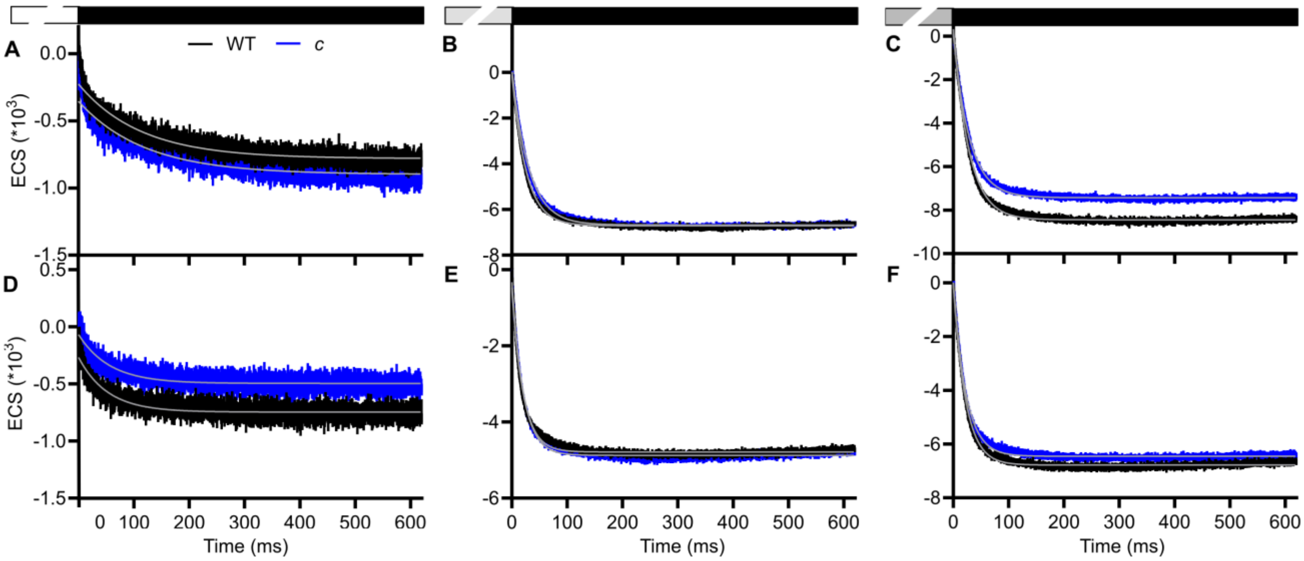

Supplementary Figure 3. Electrochromic shift (ECS) decay analysis. The wild type (WT) and *clce* (*c*) mutant plants were dark-acclimated for 30 min and then exposed for 210 s (**A**–**C**) or 15 min (**D**–**F**) to light at 15 µmol photons m^{-2} s^{-1} (**A**, **D**), 100 µmol photons m^{-2} s^{-1} (**B**, **E**) or 650 µmol photons m^{-2} s^{-1} (**C**, **F**), after which the light was switched off to record ECS during 600 ms dark intervals. The ECS decay of the first 100 ms was fitted to calculate g_{H^+} (s^{-1}) =1/time constant for decay.

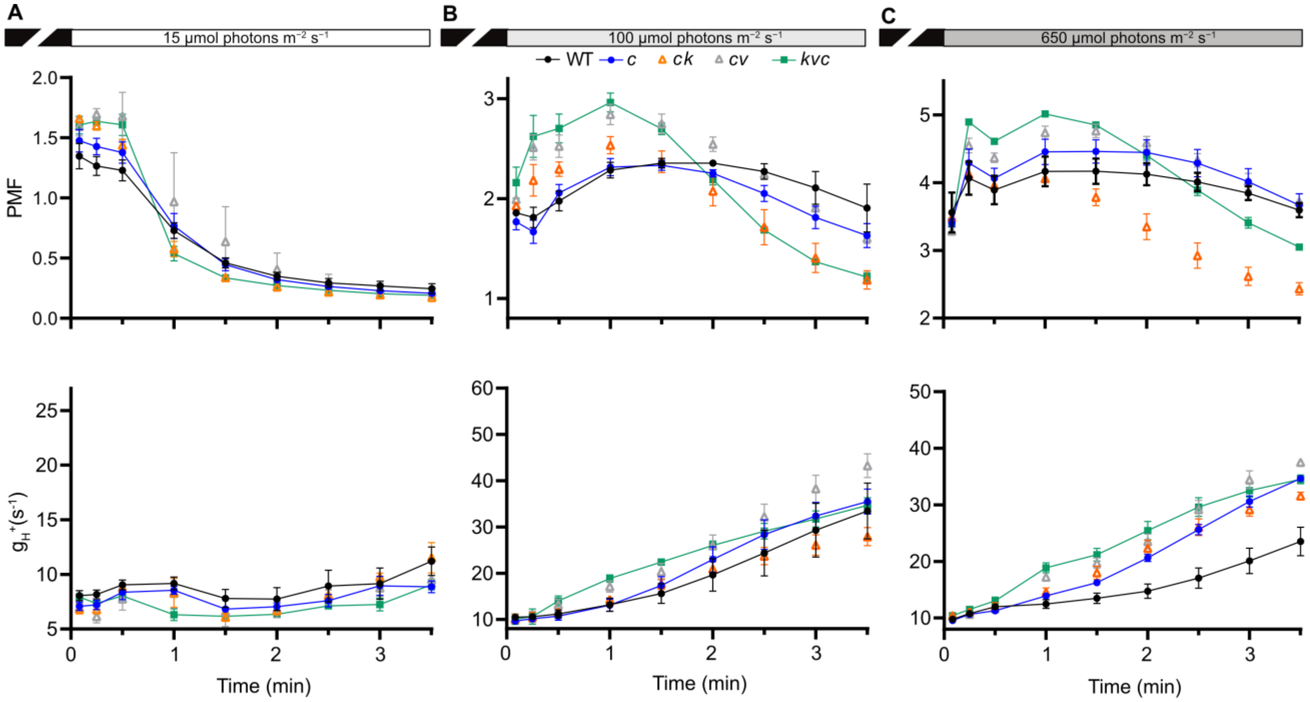

Supplementary Figure 4. Induction kinetics of proton motive force and H^+ conductivity through ATP synthase. Electrochromic shift measurements (ECS) were performed on 30 min dark-acclimated wild-type (WT) and mutant plants grown in short-day conditions (120 µmol photons m^{-2} s^{-1}) and exposed to light at the indicated intensities. Total proton motive force (PMF) and ATP synthase H^+ conductivity (g_H^+) were calculated from ECS decay kinetics as described in Methods. The plotted data are means ± S.E.M. (*n* = 6 plants). WT – *Col-0*, *c* – *clce-2*, *k*– *kea3-1*, *v* – *vccn1-1*, and *kvc* – *kea3-1vccn1-1clce-2*. Statistical analyses at 210 sec of illumination are presented in Supplementary Table 2. The kinetics for 15 min of illumination are presented in Figure 4.

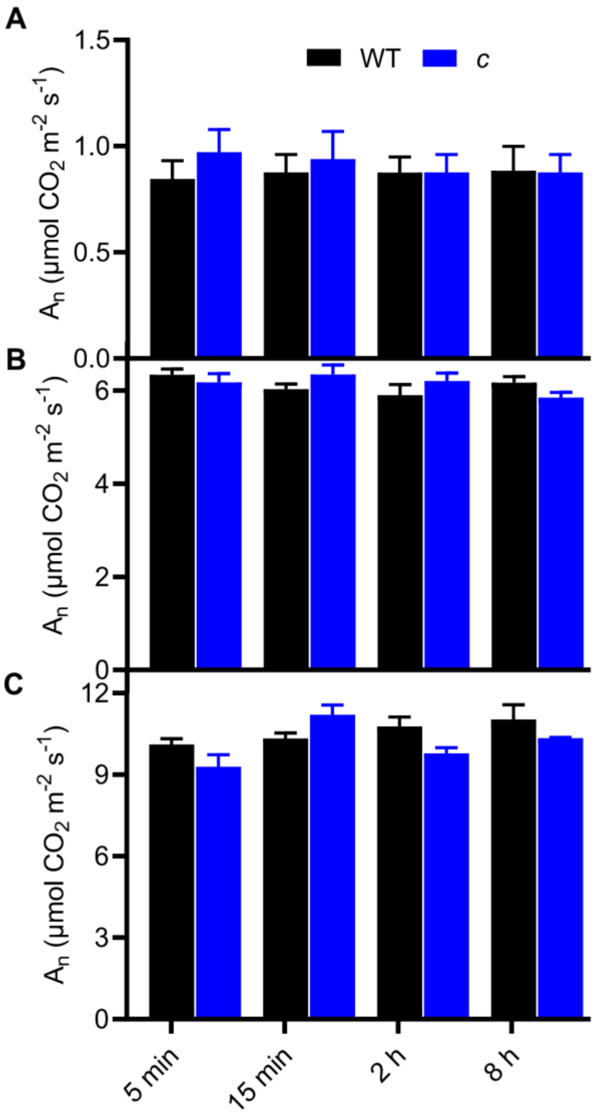

Supplementary Figure 5. Net photosynthesis. Wild type (WT) plants and the *clce* (*c*) mutant were grown using short-day photoperiod (120 µmol photons m^{-2} s^{-1}) and carbon fixation in terms of net photosynthesis (A_n) was measured at atmospheric CO_2 concentration during illumination at 30 (**A**), 150 (**B**) and 700 µmol photons m^{-2} s^{-1}(**C**) for the indicated periods of time. Data are the means ± S.E.M. (n = 3–5 plants). There were no statistically significant differences between WT and *clce* at any of the tested conditions according to Student's *t*-test (P > 0.05).

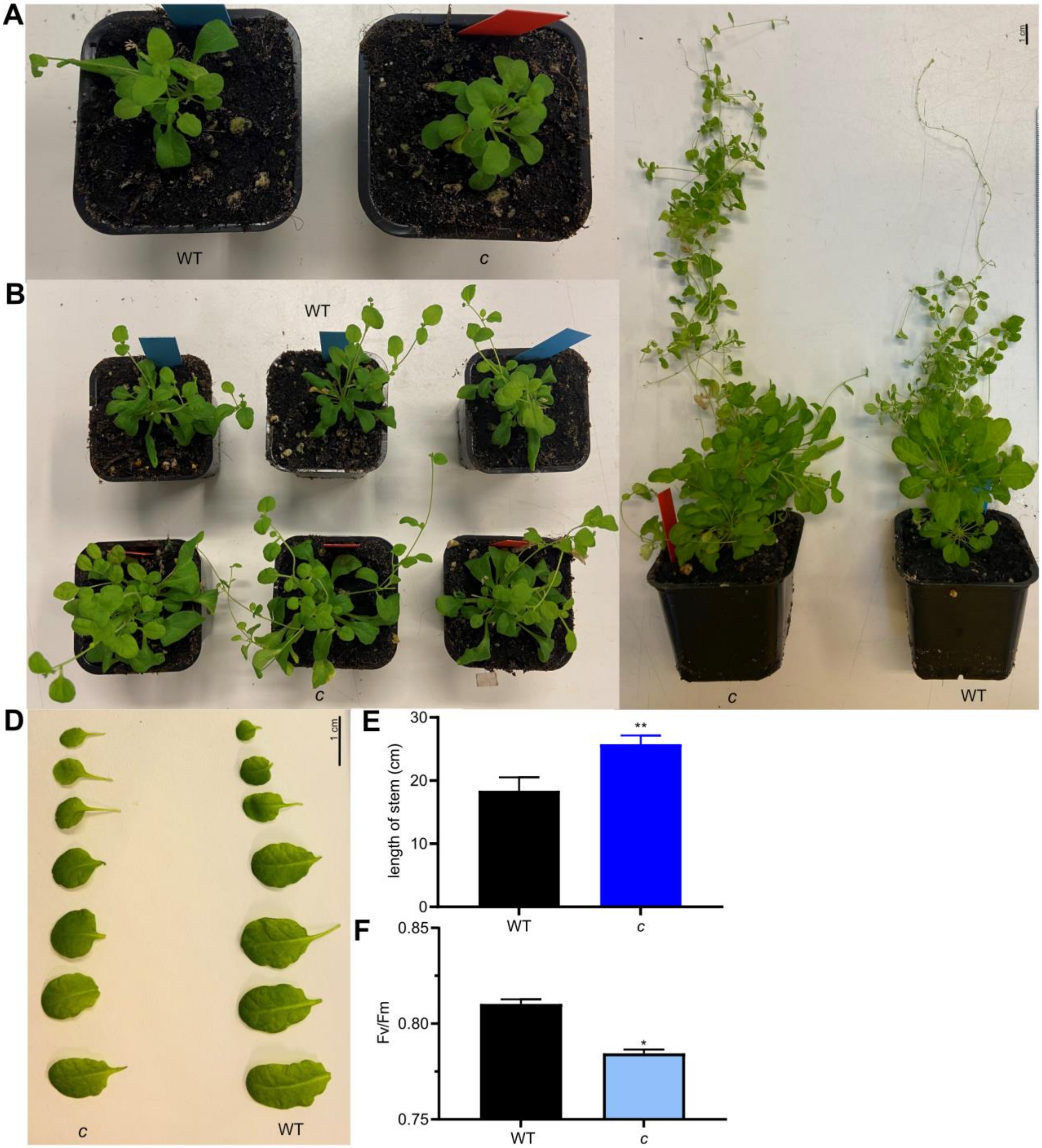

Supplementary Figure 6. Growth phenotype in low light. Wild type (WT) plants and the *clce* (*c*) mutant were grown in short day conditions with 8 h light (15 µmol photons m^{-2} s^{-1})/16 h dark for 3 months **(A),** 5 months **(B)** and 8 months **(C)**. **(D)** Leaves from 8 months-old plants. (**E**) Length of stems from 8-months-old plants. (**F**) F_v/F_m of plants grown under low light for 8 months**.** Data are the means ± S.E.M. (*n* = 3 plants). Asterisks denote a statistically significant difference between WT and *clce* according to Student's *t*-test (** - *P* < 0.01, * - *P* < 0.05).

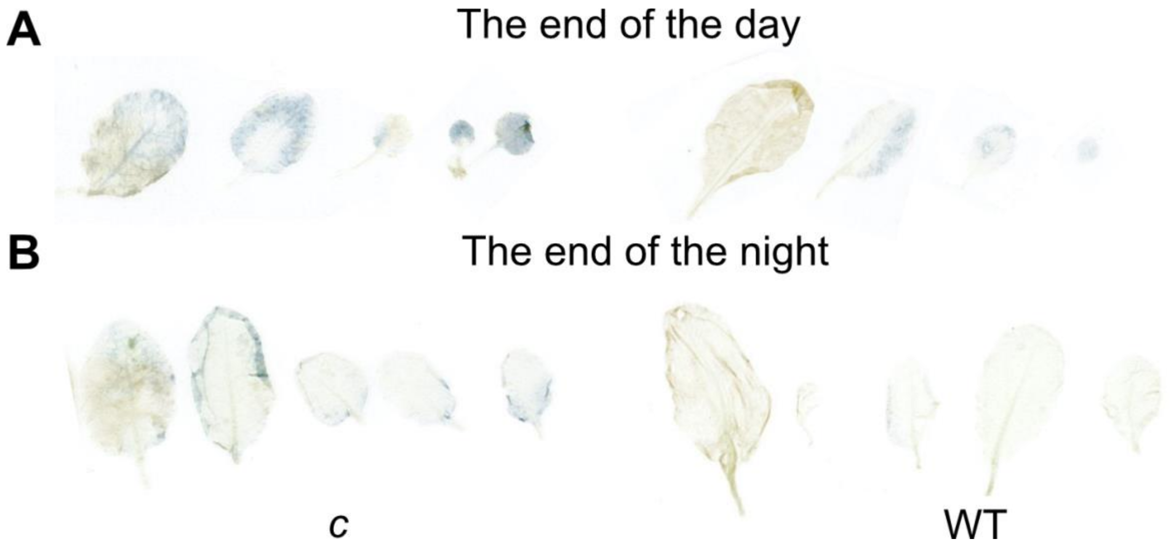

Supplementary Figure 7. Iodine-stained Arabidopsis leaves visualizing starch distribution (dark coloration). Wild type (WT) and *clce* (*c*) plants were grown in short-day conditions at 15 µmol photons m^{-2} s^{-1}. Accumulation of starch was followed by iodine staining at the end of the day (**A**) and at the end of the night (**B**).

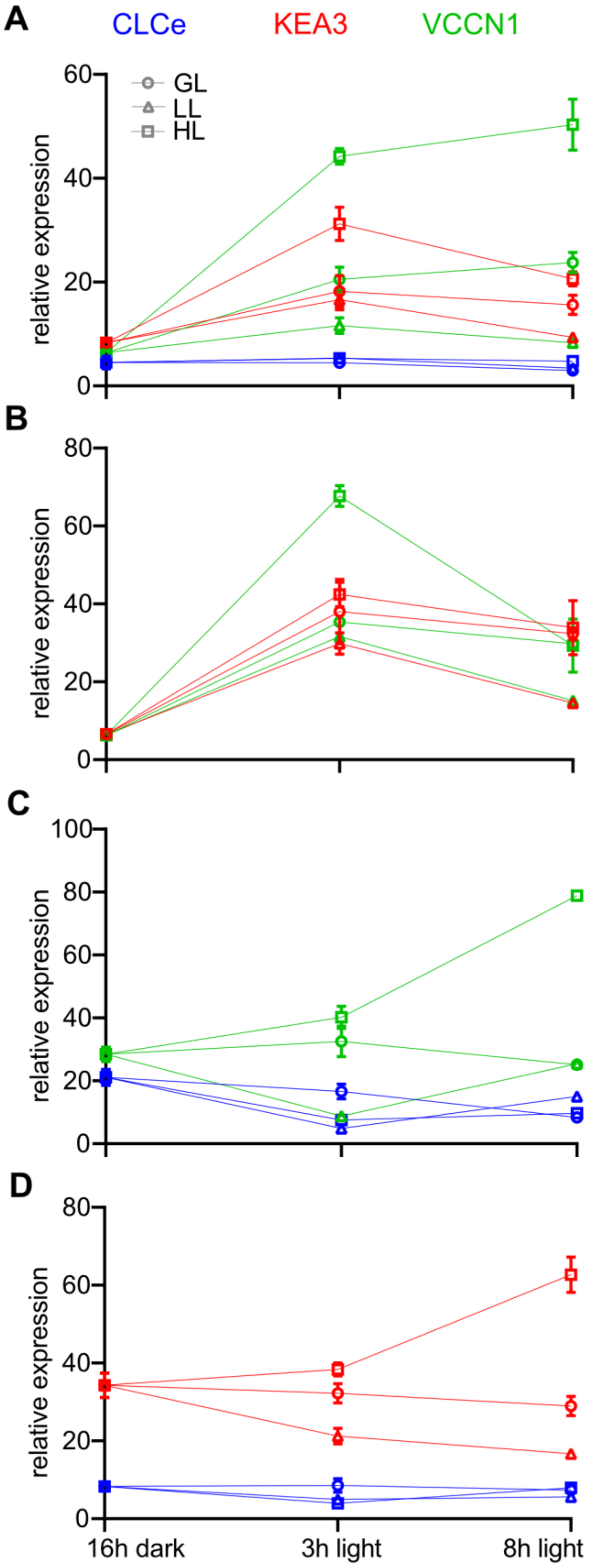

Supplementary Figure 8. Time course for the relative expression of *CLCe*, *KEA3* and *VCCN1* genes. Wild type, *clce*, *kea3* and *vccn1* plants were grown in short-day conditions with 8 h light (120 µmol photons m^{-2} s^{-1})/16 h dark for 6 weeks. Total RNA was isolated after 16-h dark, 3 h and 8 h exposure to low light (LL, 15 µmol photons m^{-2} s^{-1}), growth light (GL, 120 µmol photons m^{-2} s^{-1}) or high light (HL, 650 µmol photons m^{-2} s^{-1}), and changes in transcript abundance were determined by quantitative RT-PCR. The expression of *CLCe*, *KEA3* and *VCCN1* genes in wild type (**A**), *clce* (**B**), *kea3* (**C**), and *vccn1* (**D**) was calculated relative to two reference genes and normalized to the expression in samples collected after the 16-h of dark period. Data are the means ± S.E.M. (*n* = 4 plants).

Supplementary Tables

Supplementary Table 1. Primers used for quantitative RT-PCR in this work.

Arabidopsis gene	BioRad unique assay ID	Chromosome location	Amplicon length (bp)
CLCe (At5g06460)	qAll1CEO0053944	4:16838714-16838848	105
VCCN1 (At3g61320)	qAll1CEO0042758	3:22695306-22695425	90
KEA3 (At4g04850)	qAthCED0053303	4:2456505-2456608	75
ACTIN8 (At1g49240)	qAll1CEO0047270	1:18217942-18218083	112
PEX4 (At5g25760)	qAll1CEO0054490	5:8968102-8968288	157

Supplementary Table 2. Statistical analyses of Supplementary Figure 4.

Light intensity	Parameter	Time point	WT	*c*	*ck*	*cv*	*kvc*
15	PMF	210 s	a	a	a	a	a
15	g_H^+	210 s	a	a	a	a	a
100	PMF	210 s	a	a	b	a	b
100	g_H^+	210 s	a	a	a	a	a
650	PMF	210 s	a	a	b	a	c
650	g_H^+	210 s	a	b	b	b	b

Different letters denote statistically significant differences among genotypes according to ANOVA ($P < 0.05$). WT – Col-0, *c – clce-2*, *ck– clcekea3-1*, *cv – clcevccn1-1*, and *kvc – kea3-1vccn1-1clce-2*.
